# Stakeholder perceptions of a nurse led walk-in centre

**DOI:** 10.1186/1472-6963-12-382

**Published:** 2012-11-05

**Authors:** Rhian M Parker, Jane L Desborough, Laura E Forrest

**Affiliations:** 1 Australian Primary Health Care Research Institute, Australian National University, Level 1 Ian Potter House, Cnr Marcus Clarke and Gordon Sts, Acton, ACT, 0200, Australia; 2 Centre for Research and Action in Public Health, University of Canberra, Canberra, ACT, 2601, Australia

## Abstract

**Background:**

As many countries face primary care medical workforce shortages and find it difficult to provide timely and affordable care they seek to find new ways of delivering first point of contact health care through developing new service models. In common with other areas of rural and regional Australia, the Australian Capital Territory (ACT) is currently experiencing a general practitioner (GP) workforce shortage which impacts significantly on the ability of patients to access GP led primary care services. The introduction of a nurse led primary care Walk-in Centre in the ACT aimed to fulfill an unmet health care need in the community and meet projected demand for health care services as well as relieve pressure on the hospital system. Stakeholders have the potential to influence health service planning and policy, to advise on the potential of services to meet population health needs and to assess how acceptable health service innovation is to key stakeholder groups. This study aimed to ascertain the views of key stakeholders about the Walk-in Centre.

**Methods:**

Stakeholders were purposively selected through the identification of individuals and organisations which had organisational or professional contact with the Walk-in Centre. Semi structured interviews around key themes were conducted with seventeen stakeholders.

**Results:**

Stakeholders were generally supportive of the Walk-in Centre but identified key areas which they considered needed to be addressed. These included the service's systems, full utilisation of the nurse practitioner role and adequate education and training. It was also suggested that a doctor could be available to the Centre as a source of referral for patients who fall outside the nurses' scope of practice. The location of the Centre was seen to impact on patient flows to the Emergency Department.

**Conclusion:**

Nurse led Walk-in Centres are one response to addressing primary health care medical workforce shortages. Whilst some stakeholders have reservations about the model others are supportive and see the potential the model has to provide accessible primary health care. Any further developments of nurse-led Walk-in Centres need to take into account the views of key stakeholders so as to ensure that the model is acceptable and sustainable.

## Background

Primary care is the first point of contact within any health care system. Countries with strong primary health care systems are able to provide more cost effective health care and can achieve better health outcomes [1]. However, many countries face primary care medical workforce shortages, find it difficult to provide timely and affordable care and, therefore, seek new ways to deliver first point of contact care by increasing the utilisation of nursing and allied health professionals and reducing dependence on medical practitioners. For instance, the United States of America (USA), United Kingdom (UK) and Canada, amongst others, have opened primary care Walk-in Centres staffed by a variety of health professionals [2].

Australia is experiencing health workforce shortages across a range of health disciplines. Shortages are not uniformly distributed and vary across geographic areas and disciplines [3]. In common with other areas of rural and regional Australia, the Australian Capital Territory (ACT) is currently experiencing a general practitioner (GP) workforce shortage which impacts significantly on the ability of patients to access GP-led primary care services. The ACT has 67.2 GPs per 100,000 people compared to the current national average of 90.7 [4]. While there have been a range of strategies developed to attract more GPs to the ACT [5], innovative models of care, including the use of nurses to provide primary care services, have also been developed in response to this shortage. The introduction of a nurse-led primary care Walk-in Centre in the ACT in May 2010 is one such innovation and was modelled on Walk-in Centres in the United Kingdom (UK) [6]. The Walk-in Centre is a publicly funded service provided to patients free of charge. The Walk-in Centre, staffed solely by registered nurses, is the first of its kind to be opened in Australia and provides walk-in access to acute and episodic care in accordance with clinical protocols without the need for an appointment. The Walk-in Centre is staffed by nurse practitioners and advanced practice nurses. In Australia, nurse practitioners are educated to Masters level to work both autonomously and collaboratively in advanced and extended clinical roles [7]. Advanced practice nurses are registered nurses who have extensive experience and are highly skilled in their field of practice [8].

The Walk-in Centre is situated at The Canberra Hospital (TCH) and very near the Emergency Department (ED) of that hospital. The hospital also has an after-hours GP-led primary care clinic on site, the Canberra Afterhours Locum Medical Service (CALMS). Therefore, the implementation of the Walk-in Centre stood to impact many stakeholders including clinicians and nurses who were already practicing at the hospital site in ED, CALMS and other outpatient clinics. The evaluation of the UK Walk-in Centres found differing levels of support amongst different health professionals [9]. Hence, it is important to gauge the views of key stakeholders and opinion leaders about any innovation in health service delivery both in terms of the acceptability of the service model and for feedback on how the service is operating [10].

Stakeholders have the potential to influence health service planning and policy, to advise on the potential of services to meet population health needs and to assess how acceptable health service innovation is to key stakeholder groups [10]. Stakeholder resistance to new models of health service delivery can undermine their implementation and threaten their sustainability. Evaluation of the UK Walk-in Centres found differing levels of support amongst different health professionals [9].

### Aim

This study aimed to ascertain the views of key stakeholders about the ACT Walk-in Centre. Specifically, it sought views on Walk-in Centre systems, organisation and quality; relationships with key stakeholders; community perceptions of the Walk-in Centre and the impact of the Walk-in Centre on access to primary health care. The study was part of a comprehensive evaluation of the Walk-in Centre conducted between May 2010 and May 2011.

## Methods

The framework for the evaluation was adapted from a conceptual framework for performance assessment in primary health care (FPA_PHC) [11]. This framework utili**s**es Donabedian’s quality of care framework linking structure, process and outcome [12] and facilitates the capacity to draw links between policy, organisational structures and processes, processes of care and patient outcomes. Ethics approval to interview the stakeholders of the ACT Health Walk-in Centre was received from the ACT Health Human Research Ethics Committee (ETHLR.11.028) and subsequently given expedited approval by the Australian National University Human Research Ethics Committee (protocol no. 2011/120). Stakeholders were purposively selected through the identification of individuals and organisations which had an expressed interest in the Walk-in Centre. A total of twenty five (n=25) stakeholders were invited to participate in interviews to gauge stakeholder satisfaction with the Walk-in Centre. Information about the project and participation was sent via email. Stakeholders who wanted to participate in the project contacted the research team. Eleven stakeholders were members of the Walk-In Centre Clinical Advisory Group. This group was formed prior to the opening of the Walk-in Centre to provide a source of ongoing advice and discussion regarding the Walk-in Centre. In addition to this group, other stakeholders were identified through ACT Health documents, which identified those included in early consultation processes. Some potential participants were identified by the evaluation team at different times during the evaluation; these included emergency department nursing and medical staff and Walk-in Centre reception staff. Participation comprised a face-to-face interview and all participants gave written consent prior to interview. Interview questions were developed under the sub-headings identified within the ‘Organisational Structures and Processes’ aspect of the FPA_PHC framework: (Physical facilities and equipment; Human resources management; Information systems; Staffing; Service organisation and management; Processes of care provided; Inter-provider agency networks and relationships; Community networks and relationships; Performance assessment). These sub-headings are identified as those required to be established, implemented and maintained by primary health care providers [11]. Within these sub-headings details of questions were developed utilizing Hollander et al’s framework [13] for the evaluation of health care services. Key questions are identified determinant on the nature of the evaluation; proof of concept, implementation, process or outcome evaluation [2]. Interviews were conducted by RP, JD and LF. Interviews were audio recorded and professionally transcribed verbatim. Identifying information about the participants was removed. NVivo 8 software (QSR International Pty Ltd., Melbourne, Australia) was used as a data storage and analysis tool. Interviews were analysed by JD, who discussed the analysis and themes regularly with RP and LF, with a focus on patterns within the data and identifying common threads.

## Results

Seventeen (n=17) stakeholders agreed to participate in the study. Five of these (one group of two people and one of three people) requested to be interviewed together rather than individually. Participants included two general practitioners, a physiotherapist, a pharmacist, two consumer representatives, a hospital administrator, two hospital managers, two nurses, two health department staff, two emergency medicine clinicians, two representatives from the Australian Medical Association both of whom were medical practitioners. Those who did not respond to the invitation to participate included Walk-in Centre reception staff, representatives of the Pharmacy Guild and the Australian Nursing Federation and three members of the Clinical Advisory Group and one ACT Health clinical manager. Despite the large number of stakeholders who did not participate, the researchers believed that the final sample provided a good representation of the perspectives of a broad group of stakeholders. The variety of participants ensured feedback obtained was representative of diverse views. Disparate views were identified and explored with regard to the relationships, tensions and similarities with other data. This process ensured a rigorous approach to data analysis. Results are reported within the sub-headings identified within the design of the study.

### Walk-in Centre system, organisation and quality

#### Information systems and protocols

The Walk-in Centre uses a Clinical Decision Support Software (CDSS) to support nurses at the Centre in clinical decision making at the point of care. However, the CDSS is a standalone system that doesn’t integrate with other systems within ACT Health:

"Stakeholder 1: It is a standalone system, which we should never have standalone systems in ACT in this day and age. It doesn’t interface with our system, it doesn’t interface with Clinical Portal Concerto, so you can’t… if a patient comes into the Emergency Department 24 hours after they’ve been to the Walk-In Centre they cannot see the record of what’s happened in the Walk-In Centre. And that should never have been, in my opinion, allowed to happen …"

The protocols utilised by the Walk-in Centre nurses are perceived by stakeholders to be both a source of support and limitation for the nurses working at the Walk-in Centre. Whilst they are a valuable and supportive risk management strategy, they are perceived to limit the capacity of the nurses to utilise their experience and clinical decision making capacity in line with their scope of practice:

"Stakeholder 2: I would probably argue that because of safety and the protocols, and the way things have been set up, that more time is spent with patients than necessarily is required for the minor complaint that they’ve come for. It provides a wonderful, safe sort of care episode for the patient. I don’t know how sustainable it is that nurses will have that much time to provide that, but I think patients love it. I think with evolution those protocols will be changed, in as much as there will be greater autonomy, greater decision making ability."

#### Model of care

Most stakeholders are satisfied with the nurse-led model of care; however most stakeholders also believe that, for the model to be successful, the scope of practice of the advanced practice nurses needed to be extended and the nurse practitioners must be supported to fully implement their roles:

"Stakeholder 4: I think it’s a safe model of care. I don’t know if it uses the full scope of practice of registered nurses or advanced care nurses that are in practice, it certainly goes no way to fostering a culture of positive thought around nurse practitioners… I think it’s a shame…"

#### Quality of care

Most stakeholders’ comments regarding quality of care were anecdotal:

"Stakeholder 3: And I think that really the feedback anecdotally from people in the community is how impressed they’ve been with the service that they’ve received."

Those with professional experience of patients attending the Walk-in Centre have positive feedback:

"Stakeholder 10: And I think they have good assessment skills. There’ve been a couple of very good pickups where they’ve picked up really sick patients."

#### Cost of care

Whilst quality of care is considered high, the cost of this care is flagged as an issue to be considered:

"Stakeholder 9: The quality of care, I think, is very good. But it comes at a cost and the cost is in relation to staff, the hours of operation and the fact that for all intents and purpose it’s a free service to the client. So, the quality is good, but it does come at a cost."

There was concern amongst two stakeholders that the funds allocated to promoting the Centre were too high and could have been better utilised elsewhere in the ACT primary health care system.

#### Nurse practitioners

The role of nurse practitioners in the Walk-in Centre was raised by most stakeholders, who acknowledged that this role has not been fully implemented. This has implications for the scope of practice of the Walk-in Centre itself and subsequently the successful implementation of this nurse-led model of care. Many stakeholders are aware of the frustration experienced by nurse practitioners employed in the Walk-in Centre due to their diminished capacity to fulfill their role:

"Stakeholder 4: I guess I have a real worry that the nurse practitioner role will not progress in the Walk-in Centre and I think that will be a significant loss because I guess the nurse practitioner expertise is extremely valuable. Even at the moment the nurse practitioner working in the Walk-in Centre is not working as a nurse practitioner, but advanced practice nurse. The expertise he brings to the group is invaluable and I think if that is lost, it’ll mean that the overall care will suffer. I don’t know what the answer is?"

#### Alternative models of care

Whilst the current nursing model of care has not been fully realised, a number of stakeholders believe the model of care could be enhanced with the inclusion of a doctor. The model would still be nurse-led, with the doctor’s presence providing a source of collaboration, mentorship and referral for patients who fall outside of the scope of nursing practice:

"Stakeholder 2: I think perhaps the option for them to keep that idea that it’s a nurse led clinic would be to have the nurses assess the patients and then to bounce off a doctor, as opposed to a doctor taking in a patient …, so the nurses are still assessing patients and treating them accordingly but they’ve got that buffer. Or if that patient, or there’s one patient that’s a little bit grey in terms of which pathway to take they can say to the doctor can you finish this."

#### Staff education and training

There is general concern with the provision of training and ongoing education for Walk-in Centre staff. In particular, stakeholders involved in the initial training for Walk-in Centre staff voiced concern in regard to the provision of training for new staff, which at this point in time, some perceive to be non-existent:

"Stakeholder 6: One of the concerns I do have, we did very intense training on medicines with the new staff before the centre opened, but my concern is what’s going to happen to the new staff that are now recruited with that training."

The need for ongoing professional development was identified, in particular for nurse practitioners:

"Stakeholder 1: These are expert senior nurses, and they’re very hungry for continuous improvement, and whilst they’re delivering a service, it’s very hard for them to have quarantine time. And as well, they’re now in a model of care that’s autonomous, and so there aren’t people in the workplace who know more than them, to give them real time feedback, and that has been expressed as an area for improvement, or development."

### Relationships with stakeholders

Most stakeholders are satisfied with the relationships they have with the Walk-in Centre. The capacity for the Walk-in Centre to foster its relationships with other organisations and health care providers has been enhanced through the development of these relationships throughout the first year of operation:

"Stakeholder 5: It was a really significant amount of work, but we’ve had a very good relationship with them and they understand its work on top of what we normally do and it’s been very amicable, but I guess it could have potentially not been."

#### Relationship between the emergency department and the Walk-in Centre

There were mixed views about the relationship between the ED and the Walk-in Centre. In particular, relating to a potential for the Centre to add to the workload of the ED with patients who were outside the Centre’s scope of practice:

"Stakeholder 10: But my overall impression of how that has been is that we seem to get a lot of patients that have gone to the Walk-in Centre, so they’ve done the right thing but somehow they are referred up to us. And so it seems to add extra work in many instances."

However one stakeholder cited data providing evidence that a large number of referrals to ED are appropriate:

"Stakeholder 2: The data suggests that those referred from Walk-in Centre to ED are appropriate – 25% are triaged as Category 3 in ED."

### Community perception of the Walk-in Centre

Most stakeholders believe those in the community who are aware of the Walk-in Centre are supportive of it; consumer representatives stated there is significant support for it:

"Stakeholder 12: Well certainly my understanding is that they’ve engaged with consumers quite well. There’s a consumer representative on the Clinical Advisory Board so I think at that level there’s the opportunity for the community to be engaged. I guess we could always all engage more with the community in terms of what we’re doing so that they understand what we’re trying to achieve but I don’t see that they haven’t done that. It seems to me that they’ve done that quite well and they’ve got their frequently asked questions and their website so it appears that they have."

However, there is a significant perception that the community has not been adequately informed about the Walk-in Centre. A number of stakeholders believe ongoing and improved marketing could improve public awareness of the Walk-in Centre and of the Centre’s scope:

"Stakeholder 13: You know there’s certainly some people that do go there but most of the people that I see … they say oh what’s that, where is it? So there is still, you know they certainly could do more advertising into the community, I certainly think so."

"Stakeholder 4: The only couple of negative reports that I’ve heard have been because people have not understood the scope of practice, so they’ve been expecting a lot more and I think it needs to be clarified that the Walk-in Centre is not to become your go to every time instead of a GP place."

### Access to primary care services

In terms of general practice, the number of patients seen in the Walk-in Centre is not considered large enough to have had a noticeable impact at this stage:

"Stakeholder 13: I can’t see how, with the numbers and the volume, it could have had an impact."

Nevertheless two stakeholders believe the Walk-in Centre has had a negative impact on general practice through a loss of funds spent on the Walk-in Centre that could otherwise have been invested in general practice. Overall the Walk-in Centre is perceived by most stakeholders to have improved access to primary care; however it is perceived that this impact could be greater if the scope of practice of the staff were extended and expanded.

## Discussion

Stakeholders’ concerns about the Walk-in Centre CDSS were widespread and refer to its capacity to interface with other systems and to generate referrals and specific data reports. The use of software with well-integrated clinical decision support facilitates documentation, electronic referral and peer review [14]. Additionally, well integrated systems can enhance patient safety through sending electronic prescriptions to pharmacies and providing automated medical record and medication history checks [14]. Whilst the protocols are seen to be a valuable, supportive risk management strategy, they are perceived to limit the capacity of the nurses to utilise clinical decision-making skills, thus utilising their full scope of practice. Enablement of this would increase the capacity of the Walk-in Centre to improve access to broader primary care services for patients. Such concerns are not new and Salisbury et al. note in their evaluation of the nurse led Walk-in Centres in the UK that nurses experienced difficulties with the clinical decision support software and: *The implementation of clinical assessment software within face-to-face consultation should therefore be seen as highly experimental and subject to careful planning and ongoing evaluation* [9:130]*.*

Implementation of the nurse practitioner role was also of concern to a range of stakeholders. It was felt that full implementation of this role had not been realised and this limited the nurse practitioners’ ability to work to their full scope of practice. In Canadian nurse-led convenient care centres, nurse practitioners are integral in terms of leading clinical decision making and developing best practice, which guide scope of practice and service delivery [14]. However, it is also noted that; *autonomy is not interchangeable with isolation. Nurse practitioners have very high levels of decision-making, accountability, and responsibility, and value both autonomy and collaboration with other healthcare professionals* [14:7]. One proposed alternative for the Walk-in Centre was the inclusion of a doctor in the model of care. The presence of a medical source of ongoing education, collaboration and training has been identified as an important factor in implementing nurse practitioner roles [15,16]; however, this does not necessarily mean that a doctor needs to be physically present at the Walk-in Centre to achieve this. Concern about the educational preparation and on-going education of nurses at the Centre was voiced by staff working at the Centre and by external stakeholders. Again, this was an issue identified at the UK Walk-in Centres but which was seen to improve over time as the Centres became more established [9].

Most stakeholders reported that they were satisfied with the level of input they had and their ongoing relationship with the Walk-in Centre. However, challenges had been experienced by ED staff who viewed the Walk-in Centre as adding to their workload. There were significant tensions in this relationship and this trend was also identified in the UK for Centres co-located with hospital accident and emergency departments. However, it should be noted that the UK Walk-in Centres co-located with EDs were not nurse –led but had significant medical input [9].

The ACT has a shortage of general practitioners and access to primary care services are limited by this. There are differing views about how this issue should be addressed and these views were represented by stakeholders. Timely access and affordability were both of concern with consumers seeing the Centre as filling a niche in terms of access and affordability whilst medical organisations were highly critical of the funds that were invested in the Centre to provide free care with which general practice can’t compete, given that general practice services in the ACT often attract a patient co-payment. The view that funding allocated to the Walk-in Centre might have been better spent on existing primary care services was also a perception of health care providers in the United Kingdom, when discussing NHS Walk-in Centres [10].

### Limitations

There are some limitations to this study. A number of stakeholders did not respond to recruitment invitations and the absence of their input is a limitation. It is not clear why they decided not to respond. A second limitation is the absence of data measuring the perception and satisfaction of general practitioners practicing near the Walk-in Centre or from areas where most patients of the Walk-in Centre were drawn. The UK experience was that health professionals had differing opinions about Walk-in Centres with doctors being more critical of them and being concerned about a lack of continuity of care [10]. A third limitation is the capacity to measure the actual impact the Walk-in Centre on other health care providers. Again the UK experience was mixed with some providers reporting reduced workload and others an increased workload [10].

## Conclusion

Stakeholders were generally supportive of the Walk-in Centre but identified a number of key areas which they considered needed to be addressed. These included the service’s systems, full utilisation of the nurse practitioner role and adequate education and training. It was also suggested that a doctor could be available to the Centre as a source of referral for patients who fall outside the nurses’ scope of practice. The location of the Centre was seen to impact on patient flows to the ED and this was highly contentious. Whilst consumer support for the Walk-in Centre was positive there were concerns about how it engaged with some stakeholders and how it was promoted to the community. There were divergent views about whether the Walk-in Centre provided access to primary care at a reasonable cost to the community.

Nurse led Walk-in Centres are one response to addressing primary health care medical workforce shortages. Whilst some stakeholders have reservations about the model others are supportive and see the potential the model has to provide accessible primary health care. Stakeholder support is important if new health service delivery models are to succeed and any further development of nurse-led Walk-in Centres needs to take into account the views of key stakeholders so as to ensure that the model is acceptable and sustainable.

## Competing interests

The authors declare that they have no competing interests.

## Authors’ contributions

RP, JD and LF conducted the stakeholder interviews. JD analyzed the data in consultation with RP and LF. RP, JD and LF wrote the paper. All authors read and approved the final manuscript.

## Pre-publication history

The pre-publication history for this paper can be accessed here:

http://www.biomedcentral.com/1472-6963/12/382/prepub
